# External fixation versus volar locking plate for displaced intra-articular distal radius fractures: a prospective randomized comparative study of the functional outcomes

**DOI:** 10.1007/s10195-014-0317-8

**Published:** 2014-09-06

**Authors:** Rajeev Shukla, Ravi Kant Jain, Neeraj K. Sharma, Ravindra Kumar

**Affiliations:** 1 Department of Orthopedics, Sri Aurobindo Medical College and PG Institute, Indore, Madhya Pradesh 453555 India; 2 Central Research Laboratory, Sri Aurobindo Medical College and PG Institute, Indore, Madhya Pradesh 453555 India

**Keywords:** Distal end radius fracture, External fixation, Volar locking plate, Green and O’Brien score

## Abstract

**Background:**

The objective of the study was to compare the efficacy of external fixation and volar plating on the functional parameter of displaced intra-articular (Cooney’s type IV) distal end radius fractures using the Green and O’Brien scoring system.

**Materials and methods:**

This prospective randomized study comprised 68 patients treated with external fixation and 42 patients treated with volar locking plates. The patients were followed up at 6 months and 1 year after surgery. The assessment of pain, range of motion, grip strength and activity were assessed at each follow-up visit and scored according to the Green and O’Brien scoring system.

**Results:**

At 1 year after surgery, we observed that external fixation showed significantly better results than volar locking plates using the Green and O’Brien scores for range of motion (22.0 ± 4.77 vs 19.89 ± 5.05), grip strength (19.91 ± 5.4 vs 16.89 ± 4.4) and final outcome (87.36 ± 11.62 vs 81.55 ± 11.32). No difference was found in pain and activity between these two groups of patients. Patients aged <50 years treated with external fixation showed excellent results (final score (91.57 ± 9.01) at 1 year follow-up.

**Conclusion:**

External fixation showed superiority over volar locked plating after 1 year of surgery.

**Level of evidence:**

IV.

## Introduction

Fractures of the distal radius are common [1–3]. The increasing incidence of these injuries may be attributed to an aging population (osteoporotic fractures) and the growing participation in outdoor pursuits (higher energy fractures) [4, 5]. Whereas a large number of these fractures are managed non-operatively, the number of patients who undergo surgical management is considerable. Over the past 30 years, the surgical treatment of distal radius fracture has shifted from cast immobilization to numerous surgical options such as the use of external fixation and volar locking plates [6–9]. There are distinctive differences in these two surgical techniques and postoperative rehabilitation protocols. Previously some authors have compared volar locked plating with external fixation, but there is still insufficient evidence regarding which gives the best outcome [10–14].

In one meta-analysis which included 46 papers, with 916 patients treated by external fixation and 603 by internal fixation, the authors could find no evidence to support one treatment method over the other [15]. In another meta-analysis, a better functional outcome was observed in patients with unstable distal radius fractures treated with a volar locking plate compared with (augmented) external fixation at 3, 6 and 12 month follow-up [16].

The aim of this study was to compare the efficacy of external fixation with volar locked plating treatment strategies in displaced intra-articular (Cooney’s type IV) distal radius fractures.

## Materials and methods

This study was performed between June 2010 and May 2012 on patients with distal radius fractures who visited Sri Aurobindo Institute of Medical Sciences, Indore. The patient criteria for inclusion in this study were age >18 years without any other skeletal injury and with Cooney’s type IV fracture. Type IV distal radius fractures were diagnosed according to Cooney’s classification system. Patients with any other associated injury/fracture, bilateral distal radius fractures, open fractures of distal radius and associated head injury were excluded from the study.

**Fig. 1:**
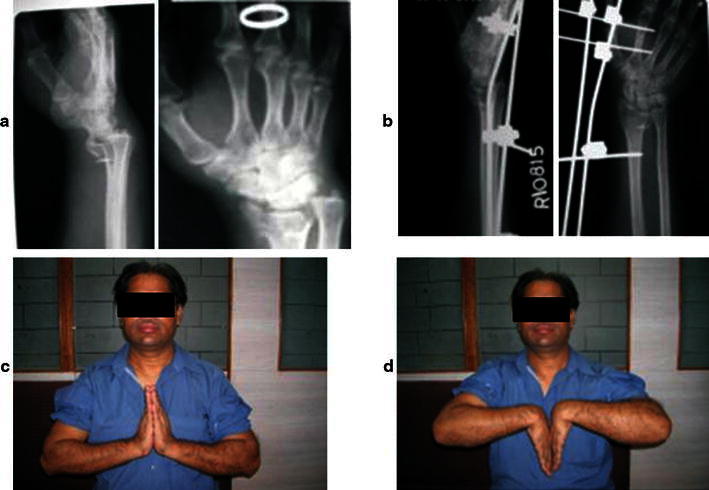
Pre- and postoperative radiographic image of the wrist of a patient in external fixation (**a,b**). Clinical picture of a patient treated with external fixation after 1 year follow-up (**c**, **d**)

The patients were randomized into two groups using random number tables generated online (http://www.graphpad.com/quickcalcs/randomize1/). The external fixation technique was chosen for group 1 and volar locking plates were chosen for group 2. All surgical procedures were performed by a single author (RS) at a single institute using standard protocols under general or regional anesthesia.The general external fixation technique used two 2.5-mm Schanz pins in the second metacarpal and two 3.5-mm pins in the radius proximal to the fracture. The pins were interconnected and tightened with solid connecting rod and link joints. After application of a frame, reduction was checked in the C-arm in antero-posterior and lateral views (Fig. 1). Reduction was achieved via manual traction and closed reduction method in all cases. Sterile betadine dressing of the pin tract site was performed. A below-elbow plaster of Paris slab was applied in all patients for 1 week. The external fixator was removed in all patients after 8 weeks. No extra wire was used in any patient since we were able to achieve reduction in fracture by use of pins only.

**Fig. 2:**
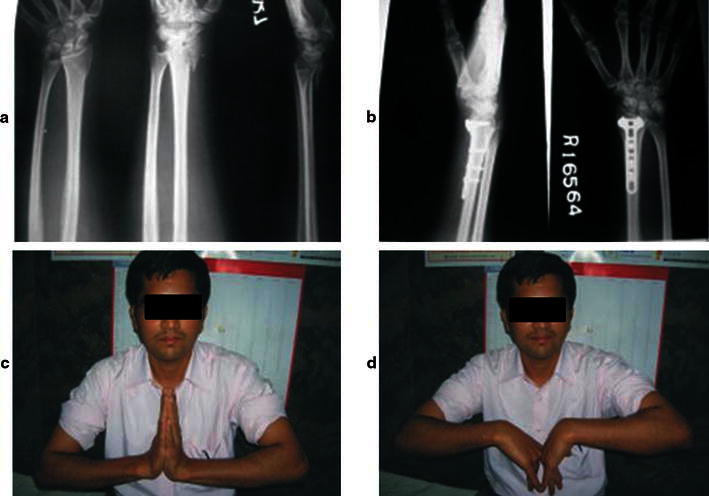
Pre- and postoperative radiographic image of the hand of a patient in volar plating (**a**, **b**). Clinical picture of a patient treated with volar plating after 1 year follow-up (**c**, **d**)

In the volar locked plating technique, the skin was incised longitudinally along the course of the flexor carpi radialis (FCR) tendon. The FCR sheath was opened and the tendon retracted to the radial side to expose the ulnar corner of the distal radius (this can be extended into a carpal tunnel release). The FCR tendon was also retracted to the ulnar side to expose the radial styloid and scaphoid fossa. Great care was taken to avoid pressure on the median nerve. Underneath the FCR sheath lies the flexor pollicis longus (FPL) tendon. This was retracted ulnarly revealing the pronator quadratus (PQ) muscle. The PQ muscle was elevated from its radial origin and reflected ulnarly to expose the distal radius. If the fracture was very distal, it was not necessary to completely elevate this muscle. The palmar extrinsic radiocarpal ligaments should not be detached from the radius to expose the joint surface as this may destabilize the wrist. Palmar fragments were often comminuted and impacted. Each fragment was identified, elevated, and reduced. As the palmar surface of the distal radius is originally flat, the application of a flat implant onto this surface usually corrects any malrotation of the fracture fragments. The C-arm was used to check for screw placement and reduction. Radiographs of the wrist joint were taken after surgery (Fig. 2). The applied casts did not allow free mobilization.

The patients of both groups were discharged 2 days after surgery after checking the suture line under proper antibiotic coverage (3rd generation cephalosporin for 3 days) and active finger movements were advised. The patients were recalled for suture removal and to see the reduction in fracture radiologically after 10 days. Acceptable criteria for fracture reduction were:

1. Radial inclination of >15°.
2. Radial shortening of <5 mm compared to the contralateral side.
3. Sagittal tilt between 15° dorsal and 20° volar tilt.
4. Intra-articular step-off of <2 mm.

All the patients were followed for 6 months and 1 year after surgery and assessed for pain, grip strength, wrist range of motion (ROM) and activity, and scored according to the Green and O’Brien scoring system. Scores <65 were considered poor, and scores between 65 and 79, between 80 and 89, and between 90 and 100 were considered fair, good and excellent, respectively.

### Statistical analysis

All data were entered in SPSS 20.0 (IBM SPSS Inc. USA). The mean values of scores between the two techniques were compared by Student’s *t*-test and scores at different intervals within the same group were compared by paired sample *t*-test.

## Results

One hundred and ten patients (61 females and 49 males) with Cooney’s type IV distal radius fractures were recruited into the study. The mean age of patients at surgery was 39.12 ± 13.06 years. There was no significant difference between groups regarding age or sex (Table 1). Follow-up data could be obtained for 109 patients after 6 months and for 100 patients (91.7 %) after 1 year. Mean surgery time was 35.1 ± 2.5 in the external fixation group and 56.5 ± 2.7 min in the volar plate fixation group. Reduction in fracture was achieved in all patients in both groups and no patient required revision surgery.

**Table 1:** Demographic profile of patients

Parameter	External fixator	Volar plate	Total	*P* value
Number	62	48	110	
Age	38.95 ± 13.15	39.33 ± 13.1	39.12 ± 13.06	0.883
Sex (male/female)	29/33	20/28	49/61	0.733

One patient in the volar locking plate group developed complex regional pain syndrome type 1 that improved within 2 months by physical therapy and pain medication. Swelling, inflammation and occasional pain were observed in two patients in the external fixation group and one patient in the volar locking plate group.

One year after surgery, 85.5 % of patients treated with external fixation and 73.3 % of patients with volar plating had an excellent or good result according to the Green and O’Brien score.

We observed a significant reduction in pain, increased ROM, grip strength, activity and final score after 1 year follow-up compared to that at 6 month follow-up in the external fixation group. In the volar locking plate group, we found there was no change in pain, ROM and grip strength; however, there was a significant change in activity and final score at 1 year compared to 6 month follow-up (Table 2).

**Table 2:** Comparison of scores after 6 months and 1 year follow-up in two treatment groups

	External fixator			Volar plate		
	6 months	1 year	*P* value	6 months	1 year	*P* value
Pain	19.91 ± 4.6	22.36 ± 2.86	0.000	21.22 ± 3.71	21.33 ± 3.5	0.570
ROM	17.36 ± 6.2	22.0 ± 4.77	0.000	19.67 ± 5.3	19.89 ± 5.05	0.570
Grip strength	16.91 ± 5.3	19.91 ± 5.4	0.000	16.78 ± 4.4	16.89 ± 4.4	0.323
Activity	21.36 ± 4.4	23.09 ± 2.6	0.000	22.67 ± 3.1	23.44 ± 2.78	0.018
Final score	75.54 ± 17.7	87.36 ± 11.62	0.000	80.33 ± 11.25	81.55 ± 11.327	0.006

Although there was no significant difference in pain, ROM, grip strength, activity and final outcome in patients at 6 months after surgery using either of these two techniques, we observed low pain and high ROM in patients treated with volar locking plates compared to those treated by external fixation (Table 3). However, at 1 year, we observed a significant increase in only ROM, grip strength and final outcome in patients treated with external fixation compared to patients treated with volar locking plates. No difference was found in pain and activity between patients in either group.

**Table 3:** Comparison of Green and O’Brien score in two techniques at 6 months and 1 year follow-up

	6 months			1 year		
	External fixator	Volar plating	*P* value	External fixator	Volar plating	*P* value
Pain	19.91 ± 4.6	21.22 ± 3.71	0.129	22.36 ± 2.86	21.33 ± 3.5	0.114
ROM	17.36 ± 6.2	19.67 ± 5.3	0.053	22.0 ± 4.77	19.89 ± 5.05	0.035
Grip strength	16.91 ± 5.3	16.78 ± 4.4	0.895	19.91 ± 5.4	16.89 ± 4.4	0.003
Activity	21.36 ± 4.4	22.67 ± 3.1	0.161	23.09 ± 2.6	23.44 ± 2.78	0.517
Final score	75.54 ± 17.7	80.33 ± 11.25	0.120	87.36 ± 11.62	81.55 ± 11.327	0.010

Patients aged <50 years treated with external fixation had a better outcome than patients aged >50 years in all parameters studied at the end of 1 year. However, in patients treated with volar plating, there was no change in pain, ROM grip strength and activity in these two age groups (Table 4).

**Table 4:** Comparison of Green and O’Brien score in two techniques at 1 year follow-up in two age groups

	External fixator			Volar plating		
	<50 years	>50 years	*P* value	<50 years	>50 years	*P* value
Pain	32.16 ± 2.4	20.59 ± 3.01	0.001	21.47 ± 3.59	19.55 ± 4.15	0.144
ROM	23.42 ± 3.6	18.82 ± 5.45	0.001	20.59 ± 5.04	17.73 ± 4.67	0.103
Grip strength	21.18 ± 4.7	17.06 ± 5.87	0.008	17.21 ± 4.47	15.0 ± 4.7	0.162
Activity	23.82 ± 2.15	21.47 ± 2.93	0.002	23.53 ± 2.62	22.73 ± 3.4	0.419
Final	91.57 ± 9.01	77.94 ± 11.46	0.000	82.79 ± 10.6	75.0 ± 9.48	0.036

## Discussion

Different types of fractures may occur due to the anatomy of the distal radius and the effects of forces in different directions. It is often not possible to have a successful outcome using the same approach and materials for different types of fractures. While mechanical characteristics are important in fixation selection, the strategic placement of the selected materials may in fact be more important than the characteristics of these materials, particularly in intra-articular fractures [17]. The best treatment option for different types of fractures may be determined by comparing different methods. External fixation is versatile in managing both intra- and extra-articular fractures with acceptable functional results. Reasons for using external fixation include the continuity of reduction under fluoroscopic control, improved reduction by ligamentotaxis, and the ability to protect the reduction until healing occurs. The advantages of external fixation are the relative ease of application, minimal surgical exposure, and reduced surgical trauma [10].

The advantages of open reduction and internal fixation include direct visualization and manipulation of the fracture fragments, stable rigid fixation, and the possibility of immediate postoperative motion. Fixed-angle plate designs minimize screw loosening in the distal fragments due to a ‘toggling effect’ and thus reduce the danger of secondary displacement. The subchondral placement of smooth pegs is useful to buttress small articular fragments and successfully control shortening and angular displacement, especially in osteoporotic bone [3]. Most fractures can be managed through a single volar access despite the presence of dorsal fragments, resulting in acceptable outcomes and good implant stability.

In the present study, 85.5 % of patients treated with external fixation and 73.3 % of patients treated with volar plating had an excellent or good result. Kapoor et al. [10] reported 80 and 63 % with good or excellent results in external fixation and volar plating groups, respectively, while Gradl et al. [17] reported 100 and 97.5 % with good or excellent results in these two groups, respectively.

As expected, higher levels of pain were observed in patients having an external fixator with the extensor tendons sliding along the distal pins. We also observed higher pain in patient treated with external fixation at 6 months after surgery, but the difference was not statistically significant.

It is thought that volar locking plates allow faster rehabilitation than external fixators. Recent prospective randomized trials have reported rapid functional recovery after volar plate application in the early period after surgery [15]. However, at 1 year, there were no significant differences between the volar locking plate and external fixator groups based on objective and subjective functional assessments [18–23]. However, Kumbaraci et al. [24] showed that the radiological and functional results of the volar plate group were better than those of the external fixator group.

Marcheix et al. [25] randomized 103 patients aged >50 years with unstable extra- and intra-articular fractures to volar locking plates. At 3 and 6 months, the plated patients had better objective functional results and reported better DASH scores, which is in accordance with our findings. The 1-year results were not reported. Wei et al. [13] compared external fixation with locked radial or volar plating and found that volar-plated patients had better DASH scores in the first 3 months. At 6 and 12 months, however, the DASH scores were similar between the groups.

In conclusion, after acceptable radiological reduction was achieved in all patients, external fixation has superiority over volar locked plating techniques at final outcome at 1 year follow-up. Patients aged <50 years had better results at the end of 1 year when treated with external fixation. Therefore we recommend external fixation technique in treating displaced intra-articular distal end radius fractures (Cooney’s type IV).
